# Editorial: High-throughput sequencing-based investigation of chronic disease markers and mechanisms, volume II

**DOI:** 10.3389/fgene.2025.1627976

**Published:** 2025-05-29

**Authors:** Hua Li, Guoshuai Cai, Wen-Lian Chen, Xiaodong Zhao, Yuriy L. Orlov

**Affiliations:** ^1^ Jiangsu Province Engineering Research Center of Development and Translation of Key Technologies for Chronic Disease Prevention and Control, Suzhou Vocational Health College, Suzhou, China; ^2^ Key Laboratory of Systems Biomedicine (Ministry of Education), Shanghai Center for Systems Biomedicine, Shanghai Jiao Tong University, Shanghai, China; ^3^ Department of Surgery, College of Medicine, University of Florida, Gainesville, FL, United States; ^4^ Longhua Hospital, Shanghai University of Traditional Chinese Medicine, Shanghai, China; ^5^ The Digital Health Center, I.M. Sechenov First Moscow State Medical University of the Ministry of Health of the Russian Federation (Sechenov University), Moscow, Russia; ^6^ Systems Biology Department, Institute of Cytology and Genetics SB RAS, Novosibirsk, Russia

**Keywords:** high-throughput sequencing, biomarker development, chronic disease, omics study, disease mechanism

## Introduction

Second-generation (short-read, massively parallel) sequencing and third-generation (long-read, single-molecule) sequencing technologies have matured rapidly, irreversibly altering how we interrogate human health and disease. A series of *Frontiers in genetics* Research Topics highlight this area (Orlov and Baranova, 2020; Anashkina et al., 2023). Building on the inaugural 2022 Research Topic (Orlov et al., 2022), this second volume of “High-throughput Sequencing-based Investigation of Chronic Disease Markers and Mechanisms” (https://www.frontiersin.org/research-topics/53085/high-throughput-sequencing-based-investigation-of-chronic-disease-markers-and-mechanisms-volume-ii/articles) again harnesses deep sequencing technologies, sophisticated analytics, and cross-scale validation to illuminate biomarkers and mechanisms that underlie a spectrum of chronic conditions - from inflammatory bowel disease to neuromuscular degeneration and pandemic infection. Together, the nine articles accepted in this Research Topic exemplify three converging trends: (i) omics integration across genome, epigenome, transcriptome and proteome; (ii) fast sequencing applications that translate into clinically actionable diagnostics; and (iii) mechanistic dissection of how candidate markers shape or signal pathophysiology.

## Gastrointestinal and metabolic diseases: decoding tissue-specific signatures

Crohn’s disease remains clinically heterogeneous and therapeutically stubborn. Yang et al. performed bulk RNA-seq of intact bowel walls and revealed two strikingly upregulated transcripts, PDE1A [OMIM 171890] and SEMA3D [OMIM 609907], associated with smooth muscle cell apoptosis and autonomic dysregulation, respectively, providing a plausible axis for the distinctive neuromuscular complications of this disease.

Turning to metabolic syndromes, Yao et al. mined public expression datasets, intersected them with ER-stress gene sets, and narrowed 49 differentially expressed genes down to three diagnostic markers - CLGN [OMIM 601858], ILF2 [OMIM 603181], IMPA1 [OMIM 602064] - that were subsequently validated in mouse models and patient sera. The study underscores how *in silico* LASSO feature selection, when wedded to wet-lab confirmation, can yield serum-accessible biomarkers for type 2 diabetes mellitus.

## Oncology: multi-omics and precise mutation discovery

Two cancer-focused articles showcase complementary high-throughput strategies. Wang et al. isolated a circulating bio-active peptide (YG-22) generated when adjuvant chemotherapy was combined with the traditional Chinese Jianpi formula; multi-layer omics (transcriptome, metabolome, chromatin accessibility, H3K4me3 ChIP-seq, NF-κB ChIP-seq) revealed that YG-22 reprograms epigenetic states and lysosomal pathways to suppress colorectal cancer cell viability. This study demonstrates the added value of peptide therapeutics derived from phytochemical regimens.

At the single-gene end of the spectrum, Zhang et al. applied targeted next-generation sequencing to four myeloproliferative-neoplasm cases that were “triple negative” by canonical testing, unmasking novel driver lesions and arguing for routine targeted sequencing in ambiguous myeloid diagnoses.

## Neuromuscular and neurodevelopmental research: from modifiers to toxicants

By pairing bulk and single-nucleus RNA-seq in healthy vastus lateralis versus tibialis anterior, Nieves-Rodriguez et al. identified >3,400 genes - including those related to calcium release and collagen-containing extracellular matrix transcripts - that may dictate differential vulnerability of muscles in Duchenne muscular dystrophy, supplying an invaluable reference for stratified gene-therapy design.

Li et al. then leveraged whole-exome sequencing of 113 patients with intellectual disability to uncover a novel *de novo* SYNGAP1 [OMIM 603384] splice-site variant (c.664-2A>G). Minigene assays confirmed exon 7 skipping, emphasizing that modest intronic changes that are detectable by high-depth sequencing can produce profound neurodevelopmental phenotypes.

Complementing human genetics, Lyu et al. used comparative transcriptomics in zebrafish embryos to show that extremely small iron-oxide nanoparticles (ESIONPs) perturb neuro-muscular development and trigger ferroptosis. Weighted gene co-expression network analysis (WGCNA) pinpointed stage-specific hub genes (highly connected nodes in the network), such as neurodevelopmental regulators and oxidative-stress mediators, whose dysregulation, together with elevated apoptosis markers, signals potential health risks of nanoparticle biomedical imaging.

## Infection and immunity: from viral alternative polyadenylation to host GWAS loci

The interface between host gene regulation and pathogen assault is another recurring theme. Tan et al. profiled grass-carp cells during early grass carp reovirus infection and uncovered extensive shifts in alternative polyadenylation (APA) despite stable DNA methylation patterns, particularly affecting cytoskeletal and microtubule genes - an underappreciated layer of post-transcriptional control in fish viral pathogenesis.

On the human front, Loktionov et al. genotyped 10 GWAS-significant loci in nearly 800 Russians and confirmed that the SLC6A20-LZTFL1 rs17713054 risk allele magnifies severe COVID-19 particularly in obese, low-activity, or low-dietary-fruit subgroups, with concordant effects on thrombodynamics. Network analyses further highlighted interactive SNP constellations linking coagulation and immune genes. Such population-targeted validation of multi-locus risk underlines the translational scope of sequencing even after the acute pandemic phase.

## Methodological cross-talk and shared biological threads

The field of gene expression regulation including chronic disease markers has been covered in a Frontiers in Genetics Research Topic (Anashkina et al., 2023) based on omics data integration. Sequencing technologies give background for gene expression regulation studies at genome scale (Anashkina et al., 2021; Orlov et al., 2023).

Across the current Research Topic, several common methodological themes emerge. First, multi-omics integration - whether combining peptidomics with chromatin readouts, or pairing methylome and APA maps - magnifies biological signals and reveals underlying mechanisms. Second, targeted or panel-based sequencing continues to sharpen genetic diagnosis where standard assays falter. Third, bioinformatics methods (WGCNA, LASSO, SNP-SNP interaction models) distill high-dimensional data into clinically tractable results.

Biologically, six recurrent pathways unite otherwise disparate studies: ER stress, calcium homeostasis, apoptotic regulation, extracellular-matrix remodeling, innate immune activation, and ferroptosis. This convergence reinforces the idea that chronic diseases, despite tissue specificity, share certain conserved response architectures that are captured by high-throughput sequencing.

## Outlook

Together, the nine articles in this volume broaden the map of chronic-disease markers, bring sequencing into daily clinical applications, and deepen our grasp of how genetic and epigenetic patterns drive long-term illness. Looking forward to this Research Topic development, we may anticipate:

• Single-cell and spatial omics will dissect cell type-restricted marker function within complex tissues such as muscle, gut, and tumor microenvironments.
• Long-read platforms will resolve structural and splice isoform diversity.
• Prospective, multi-center cohorts integrating more data (e.g., diet, exercise) with host genetics, as illustrated in the COVID-19 study, will refine gene-environment risk algorithms.
• In addition, from the current perspective, AI applications will get a more important role in complex disease studies (Koshechkin et al., 2022; Zhang et al., 2024).
